# *SigTree*: A Microbial Community Analysis Tool to Identify and Visualize Significantly Responsive Branches in a Phylogenetic Tree

**DOI:** 10.1016/j.csbj.2017.06.002

**Published:** 2017-07-06

**Authors:** John R. Stevens, Todd R. Jones, Michael Lefevre, Balasubramanian Ganesan, Bart C. Weimer

**Affiliations:** a Department of Mathematics and Statistics, Utah State University, Logan, UT, USA; b Department of Economics, Cornell University, Ithaca, NY, USA; c USTAR Applied Nutrition Research Team, Department of Nutrition, Dietetics, and Food Science, Utah State University, Logan, UT, USA; d Western Dairy Center, Department of Nutrition, Dietetics, and Food Science, Utah State University, Logan, UT, USA; e School of Veterinary Medicine, University of California, Davis, CA, USA

**Keywords:** Microbial informatics, Phylogenetic tree, Microbial community analysis, Microbiome

## Abstract

Microbial community analysis experiments to assess the effect of a treatment intervention (or environmental change) on the relative abundance levels of multiple related microbial species (or operational taxonomic units) simultaneously using high throughput genomics are becoming increasingly common. Within the framework of the evolutionary phylogeny of all species considered in the experiment, this translates to a statistical need to identify the phylogenetic branches that exhibit a significant consensus response (in terms of operational taxonomic unit abundance) to the intervention. We present the R software package *SigTree*, a collection of flexible tools that make use of meta-analysis methods and regular expressions to identify and visualize significantly responsive branches in a phylogenetic tree, while appropriately adjusting for multiple comparisons.

## 1 Introduction

The 16S rRNA gene is found in all bacteria, and 16S rRNA sequencing is one of the high-throughput genomic methods that can be used to both identify bacteria found in a sample, as well as quantify the abundance levels of individual bacterial species [1]. This is a critical element of microbial community analysis, and can shed light on complex bacterial community membership under various environmental conditions. Each bacterial species can be identified by its operational taxonomic unit (OTU) identifier, and evolutionary relationships among OTUs can be visualized in a phylogenetic tree.

It is becoming increasingly common to examine the effect of a treatment intervention (or change in environmental conditions) on the relative abundance levels of hundreds or even thousands of OTUs simultaneously in a given system. Recently published examples in the field of environmental microbiology include a study of hundreds of OTUs at each of four sites corresponding to four time points since deglaciation [2], and a study of hundreds of OTUs in soil sediment samples under different levels of aerobic methane oxidation [3]. Rather than only considering the treatment effect on the relative abundance levels of individual OTUs, it is frequently of interest to identify (and subsequently visualize) branches of a phylogenetic tree whose member OTUs exhibit a significant consensus abundance response to the treatment. That is, branches can be identified where there is sufficient statistical evidence that the *overall* OTU abundance response to the treatment is in the same direction (up or down) for the OTUs in the branch, even if some OTUs in the branch show the opposite (or no) direction response. The tree visualization tools available in *FigTree* [4], among others, can be enhanced using the significance results obtained with the software package *SigTree*, written for the R environment [5], which we present in this article. We briefly present two examples as case studies, focusing on their general demonstrative nature rather than specific biological conclusions.

The first example involves the mouse gut microbiome. Ten mice were randomly assigned to two diets (whole wheat or refined wheat), and the abundance of each of over 500 OTUs of interest was measured using 16S rRNA sequencing. (All animal procedures were performed with strict adherence to animal welfare guidelines and with oversight and approval by the Institutional Animal Care and Use Committee at Utah State University; see Methods Section 2.3 for additional study details). For each OTU, a Wilcoxon rank-sum test [6] compared the OTU's abundance in the two diets.

The second example involves cheese microbial ecology. In a repeated measures design (across 4 time points), two replicates were obtained under each of two growing conditions (the presence or absence of the probiotic bacteria Bif-6), with replicates taken from one of two cheese batches. In each replicate, the abundance of each of nearly 8700 OTUs was measured using the G2 PhyloChip; approximately 1450 of these were of interest. For each OTU, a repeated measures model was fit using the R package *limma* [7], with a contrast testing the mean abundance difference between the presence and absence of Bif-6.

## 2 Methods

### 2.1 Statistical Methods

When multiple studies of the same effect have been conducted, the field of meta-analysis [8] provides statistical methods to combine the results of those studies to arrive at a clearer understanding of the effect in question. In microbial community analysis experiments such as the mouse and cheese examples considered here, the same treatment effect has been tested in multiple OTUs, and so meta-analytic tools can be applied to the OTU-level results to arrive at a clearer understanding of the treatment effect in specific families of OTUs (or branches of the phylogenetic tree). For purposes of generalizability and flexibility, we focus on meta-analytic methods that combine the significance results (or p-values) from the OTUs [9]. Meta-analytic methods that employ results other than p-values (such as effect size estimates) could be applied, but their application would require modification for each experiment, while methods using only p-values allow for an approach fully generalizable to any experimental design, as will be shown.

In both the mouse and cheese examples, interest lies in determining which OTUs (and their member branches in the phylogenetic tree) are specifically more abundant or less abundant in the treatment group (T: whole wheat diet or Bif-6 presence) than in the control group (C: refined wheat diet or Bif-6 absence), rather than just calling OTUs (and member branches) differentially abundant. For this reason, in testing the null hypothesis μ_T_ = μ_C_ (where μ_x_ is the mean abundance of the OTU in group x), the alternative hypothesis is of the form μ_T_ > μ_C_, thus producing a one-sided p-value from the respective test (for each OTU). As a result, very small p-values (close to 0) represent evidence that treatment induces *greater* abundance than does control, while very large p-values (close to 1) indicate *less* abundance in treatment versus control.

The use of one-sided *p*-values is less common than two-sided, and deserves an additional note here regarding interpretation. Let ${\overset{-}{Y}}_{x}$ be the sample mean abundance for a given OTU in group *x*. If a one-sided test of null μ_T_ = μ_C_ vs alternative μ_T_ > μ_C_ produced a p-value of 0.01, then the corresponding two-sided test of μ_T_ = μ_C_ vs alternative μ_T_ ≠ μ_C_ would produce a p-value of 0.02 (with ${\overset{-}{Y}}_{T}>{\overset{-}{Y}}_{C}$). Similarly, a one-sided p-value of 0.99 also corresponds to a two-sided p-value of 0.02 (but with ${\overset{-}{Y}}_{T}<{\overset{-}{Y}}_{C}$). For this reason, to control the type I error rate at α = 0.05, the one-sided test requires a p-value less than 0.025 to conclude significant evidence of “μ_T_ > μ_C_”, and a p-value greater than 0.975 to conclude significant evidence of “μ_T_ < μ_C_”. This is equivalent to performing a two-tailed test, and (when the p-value is less than 0.05) concluding “μ_T_ > μ_C_” when ${\overset{-}{Y}}_{T}>{\overset{-}{Y}}_{C}$, and “μ_T_ < μ_C_” when ${\overset{-}{Y}}_{T}<{\overset{-}{Y}}_{C}$. In addition, within the one-sided test framework, if the direction of the alternative hypothesis μ_T_ > μ_C_ were switched to μ_T_ < μ_C_, the one-sided p-value of 0.01 would simply be transformed to 0.99, and the conclusion (regarding direction of effect) would be unchanged. The *SigTree* package function *p2.p1* converts two-sided p-values to one-sided p-values, given the corresponding ${\overset{-}{Y}}_{T}-{\overset{-}{Y}}_{C}$ difference (or its sign).

The most meaningful meta-analytic method to combine the one-sided *p*-values of all OTUs in a given branch is Stouffer's method [9], [10], which converts the one-sided p-values to standard normal variates, the weighted sum of which is taken as a standard normal test statistic. Briefly, if one-sided p-values p_1_, …, p_k_ correspond to k OTUs in a given branch, these are transformed to standard normal deviates Z_1_, …, Z_k_, where Z_i_ is the value in the standard normal distribution with upper-tail area p_i_. Then a statistic $\mathrm{ZS}=\sum_{i=1}^{k}Z_{i}/\sqrt{k}$ is calculated, and the Stouffer method's p-value is the upper-tail area from Z_S_ in the standard normal distribution [10].

Stouffer's method thus produces a single one-sided p-value for the entire branch, essentially corresponding to a test of H_0_: “the OTUs in the branch have no consensus of differential abundance” vs. H_a_: “there is a consensus (in a particular direction) of differential abundance in the branch.” The same directional interpretation as in the single-OTU p-value applies to this branch-level p-value (i.e., a p-value close to 0 suggests branch consensus of greater abundance in treatment, while a p-value close to 1 suggests branch consensus of less abundance in treatment). Among meta-analytic p-value combination methods, Stouffer's method has been shown to have the most meaningful “consensus” biological interpretation [11], and also favorable statistical properties (appropriate Type I error rate control, with higher power and better precision than competing methods) [12].

An implicit assumption of Stouffer's method is the independence of p-values to be combined within a given set of tests (such as a branch of the phylogenetic tree). While it could be argued that this assumption might be reasonable in some sense (as the p-value for a given OTU is obtained using only the abundance data for that OTU), there is the potential for some biologically-based dependence. Specifically, it is possible (and in some cases, perhaps to be expected) that more closely related OTUs will respond more similarly to the treatment intervention, resulting in more similar p-values for more closely related OTUs. We test for this type of dependence with permutational multivariate analysis of variance using distance matrices [13], [14], based on the adonis test of the R package *vegan* [15]. Briefly, this approach as implemented in the *SigTree* package uses a permutation test to evaluate whether the OTU p-values are independent, or whether differences among the OTU p-values are associated with between-OTU distances. “Distance” here is phylogenetic distance as represented in the corresponding phylogenetic tree.

A generalized version of Stouffer's method that allows for dependence of p-values was constructed by Hartung [16]. Briefly, and using the same notation as above for the summary of the Stouffer method, the Hartung method calculates ${\hat{\rho }}^{*}=\max\left\{\frac{-1}{k-1},\hat{\rho }\right\}$, where $\hat{\rho }$ is one minus the sample variance of the Z_1_, …, Z_k_. Then a statistic $Z_{H}=\frac{\sum_{i=1}^{k}Z_{i}}{\sqrt{k+\left(k^{2}-k\right)\left({\hat{\rho }}^{*}+0.2\sqrt{2\left(1-{\hat{\rho }}^{*}\right)/\left(k+1\right)}\right)}}$ is calculated, and the Hartung method's p-value is the upper-tail area from Z_H_ in the standard normal distribution [16].

While Hartung's method assumes a constant correlation among all pairs of p-values, its results have been shown to be stable even in the presence of a non-constant correlation [16], which would be the case in the event of a significant adonis test. Accordingly, when the adonis test indicates significant dependence among p-values in the phylogenetic tree, we recommend employing Hartung's meta-analytic method to obtain a p-value for each branch.

Because of the potentially thousands of OTUs and branches being tested simultaneously, adjustments for multiple comparisons are included in *SigTree*. The default is to control the strong family-wise error rate (FWER) using the Hommel p-value adjustment method [17], but other options exist, including p-value adjustment for false discovery rate (FDR) control while allowing positive dependence among the many tests [18]. For both error rates, the *SigTree* package converts the one-sided p-values to two-sided p-values, applies the p-value correction, and converts back to one-sided adjusted p-values so that directional interpretation is preserved.

Once the (usually error-rate-adjusted) branch-level p-values have been obtained, the *SigTree* package assigns a color (based on p-value range) to each branch and tip in the phylogenetic tree to aid in visualization. To make this visualization flexible, *SigTree* can take user-defined colors and p-value thresholds, and also export the phylogenetic tree in a Nexus format file [19], with branch colors and p-values embedded via regular expressions [20] since this format is simply a structured text file. This Nexus file is then read by tree visualization programs such as *FigTree* [4]. *SigTree* can also send the p-value and OTU members for each branch to a spreadsheet file so that significant changes in the community membership can be noted and connected to the community and the treatments in a causal association.

### 2.2 Simulation Study

A simulation study was conducted to evaluate the performance of the *SigTree* approach. No simulation could reasonably cover all possible scenarios of tree structure and treatment effects, and the underlying meta-analytic and statistical methods employed by *SigTree* have been previously validated in the literature [8], [10], [11], [12], [13], [14], [16]. Instead, the purpose of this simulation was to serve as a proof of principle that the *SigTree* approach achieves its intent within the context of phylogenetic trees, and that its results behave as expected in terms of power and type I error rate control.

**Fig. 1:**
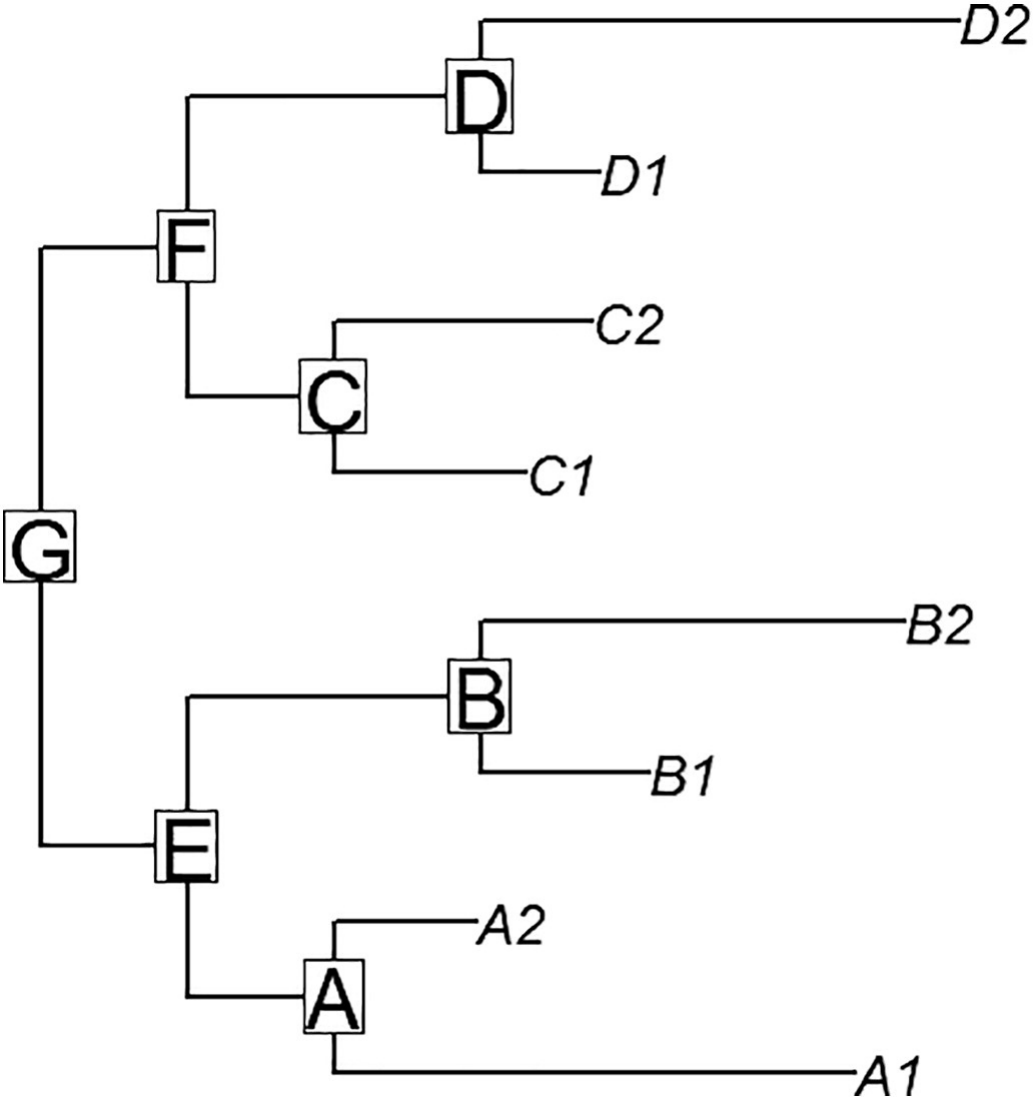
Tree outline used in simulation study.

Fig. 1 shows the basic tree outline considered in this simulation study. There are four main subtrees (or branches) – subtree A includes OTUs with a positive response to the treatment, subtree C includes OTUs with a negative response to the treatment, and subtrees B and D include OTUs with no response to the treatment. The numbers of OTUs in subtrees A and C were set at 20 and 10, respectively, and the numbers of OTUs in subtrees B and D varied from 2 to 100 (but with the same numbers of OTUs in both B and D). Fig. 1 only shows two OTUs in each subtree, just for convenience in visualization.

This simulation study assumed the relative abundance of each OTU was measured on each of 20 subjects, under both treatment and control conditions. For all OTUs, abundances in control conditions were simulated from a standard Normal(0,1) distribution. For each OTU exhibiting a response to treatment (as in subtrees A and C of Fig. 1), the magnitude of response was randomly chosen as a uniform value between 0.1 and δ, and OTU abundance in the treatment condition was simulated as a normal variate with that magnitude mean, and variance 1. The value δ varied (in ten steps) from 0.1 to 2, and the number of OTUs in subtree B (nB, same as the number of OTUs in subtree D) varied (also in ten steps) from 2 to 100. At each of the 100 combinations of δ and nB values, over 500 trees (with corresponding subject-level data) were simulated, and a *t*-test was used to obtain a raw one-sided p-value for each OTU in each simulation. The *SigTree* method was applied to each tree, obtaining Stouffer-combined and Hommel-adjusted p-values for each of the labeled nodes A–G in Fig. 1. The power and type I error rates (at each combination of δ and nB values) were assessed by taking the proportions (across simulations) of significant resulting p-values. (Here “significance” includes the appropriate direction, up or down, in subtrees A and C, respectively. The same direction could be detected in subtree E as in subtree A, and in subtree F as in subtree C, depending on the relative sizes of the subtrees and magnitude of possible treatment effect.) The type I error rate was assessed by the proportion of significant p-values in subtrees B and D, where there was no true treatment response.

### 2.3 Example Study Designs

The mouse gut microbiome study was approved as IACUC #1423, and involved individually housed C57BL/6J mice. DNA was isolated from cecal samples. The V1 + V2 125 region of the bacterial 16S rRNA gene was amplified using tag-encoded primers for pyrosequencing (Roche 454 GS FLX, Branford, CT). The V1-forward primer was 5′-AGAGTTTGATCCTGGCTCAG (BSF8) and the V2-reverse primer was 5′-CTGCTGCCTYCCGTA (BSR357). Sequencing was done at the Utah State University Center for Integrated BioSystems core sequencing facility. The representative sequences were aligned with PyNAST [21] and a phylogenetic tree was constructed with FastTree [22] after the aligned sequences were filtered with the default lanemask file and the chimeras were removed.

Microbiota sequences were processed through the data analysis pipeline QIIME [23]. Sequences were clustered into operational taxonomic units (OTUs) at a 97% sequence similarity with UCLUST [24].

In the cheese microbial ecology study, Bif-6 is the trademark name of a probiotic bacterial culture of *Bifidobacterium lactis* (Cargill Inc., Milwaukee WI). Sample collection and DNA sequencing followed protocols described in more detail elsewhere [30] and the same facilities conducted the DNA sequencing on the same equipment. For the Phylochip data, a phylogenetic tree in Nexus format of the 8700 OTUs was constructed from available taxonomical information prior to testing and visualization inside *SigTree*.

## 3 Results

### 3.1 Simulation Study Results

**Fig. 2:**
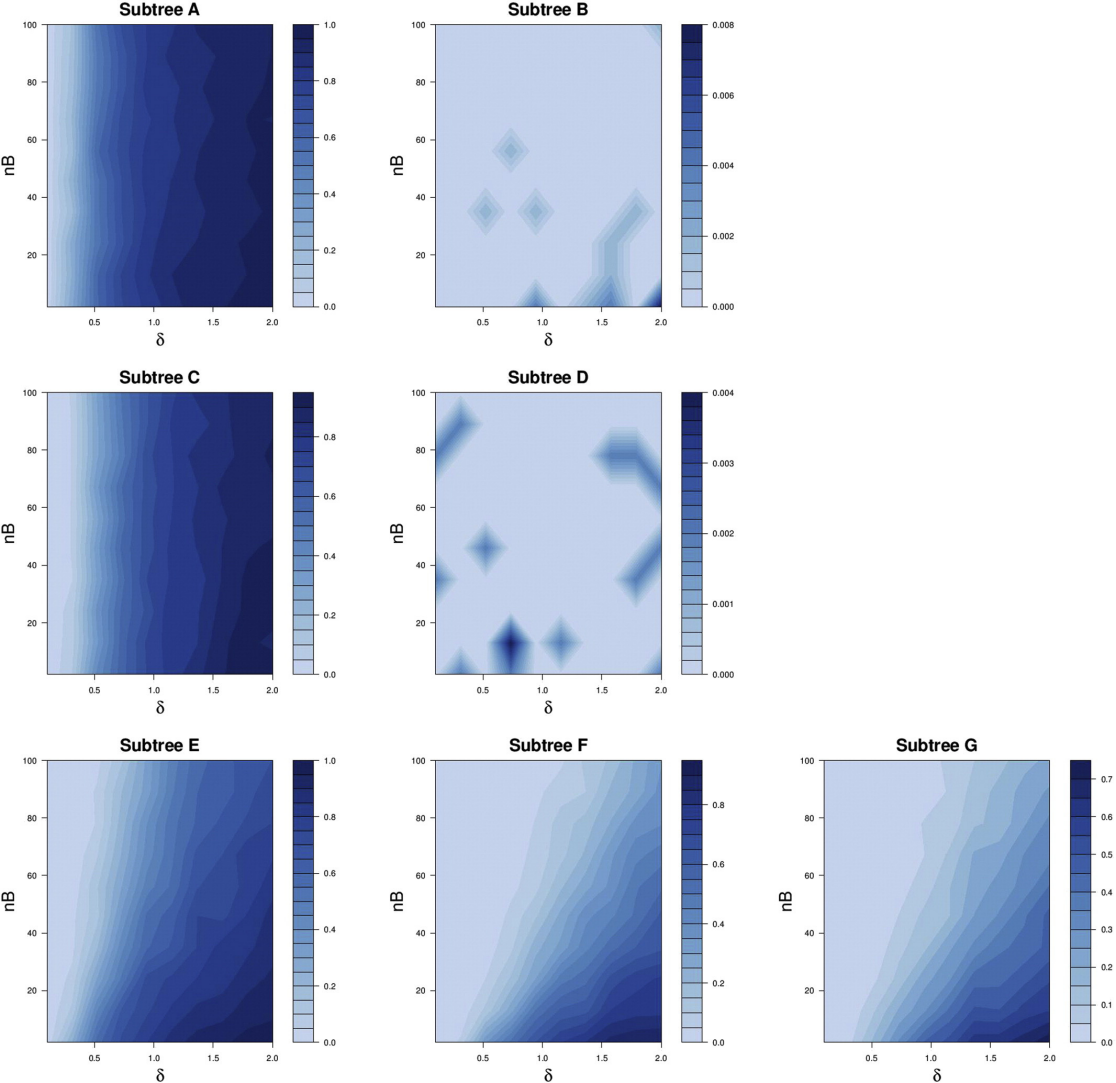
Contour plots of proportion of simulations returning significant *SigTree* results, for each lettered subtree in Fig. 1. nB is the number of OTUs in subtree B (same as number in subtree D), and δ is the magnitude of maximum possible response in subtrees A and C.

Fig. 2 shows as a contour plot the proportions of simulations where the *SigTree* approach detected significant consensus response in the lettered subtrees of Fig. 1, when the family-wise error rate was to be controlled at level α = 0.05. As would be expected, the proportions (or statistical power) steadily increase with δ for subtrees A and C, unaffected by nB (the sizes of subtrees B and D).

For subtrees B and D, which have no OTUs with a true response to treatment, the proportion of simulations where *SigTree* detects a significant consensus response would correspond to a type I error rate. Fig. 2 shows this error rate to be less than 0.01 for all combinations of nB and δ, indicating conservative type I error rate control by *SigTree*, at least for this tree structure and treatment effect framework.

For subtrees E–G, the proportions of simulations detecting significant consensus response increases with δ (possible magnitude of treatment effect), but less so as nB (the size of subtree B, as well as D) increases. Essentially, as the sizes of the “null” subtrees (B and D here) increase, their lack of any response to treatment will eventually drown out the consensus response in subtrees A and C, so that the higher-level subtrees E–G are more rarely detected as exhibiting a significant consensus response. However, for larger magnitude response (δ) in subtree A, when subtree A is a more dominant presence in subtree E (i.e., when nB is smaller), there is greater evidence of a consensus response in subtree E. This same pattern is seen in subtree F (regarding the relative size of subtree C therein), and speaks to the general interpretation of a consensus response.

While limited in scope to this one basic tree structure and treatment effect framework, this simulation study (as a proof of principle) demonstrates the expected performance of *SigTree* – appropriate power increase as the magnitude of treatment response increases (in subtrees exhibiting a consensus response), and appropriate (though possibly conservative) control of the type I error rate.

It should be noted that these simulations required nearly 6 days' worth of computation time, reduced to less than 5 hours real time, thanks to the batch computing resources of the Center for High Performance Computing at the University of Utah.

### 3.2 Example Study Results

**Fig. 3:**
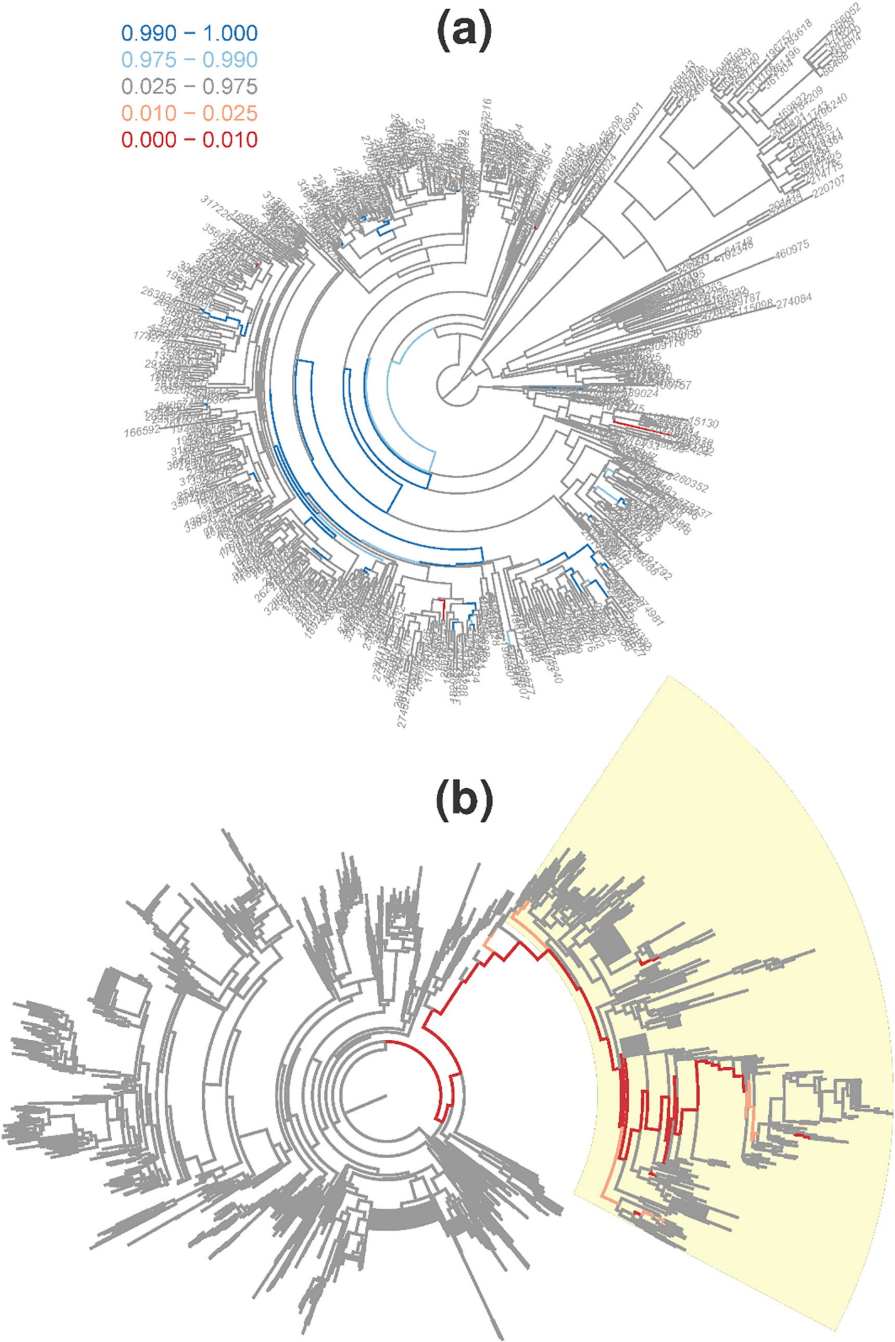
Visualization of significantly responsive branches. *SigTree* visualization of the (a) mouse gut microbiome and (b) cheese ecology phylogenetic trees, with legend for both trees showing one-sided p-value ranges on a color scale. Darker red indicates branches of OTUs more abundant in whole wheat diet than refined wheat (a), and more abundant in the presence of Bif-6 than the absence (b). Darker blue indicates branches less abundant in whole wheat (a). (For interpretation of the references to color in this figure legend, the reader is referred to the web version of this article.)

Fig. 3a visualizes the *SigTree* package results from the mouse gut microbiome example, with the FWER across the entire phylogenetic tree controlled at 0.05. (See Section 2.1′s discussion of one-sided p-values regarding why the lower and upper thresholds of 0.025 and 0.975 are appropriate here when controlling the error rate at 0.05.) Hartung's method was used to obtain the branch-level p-values, as the adonis test showed significant evidence (p-value 0.0012) of distance-based dependence among the OTU-level p-values. The FWER was controlled across the entire phylogenetic tree using Hommel's p-value adjustment method. A substantial portion of the phylogenetic tree (indicated by blue branches) is found to demonstrate a consensus decreased abundance in whole wheat diet compared to refined wheat diet. Because the FWER is controlled at 0.05, the probability of such a conclusion (for any given branch in the tree) being false is less than 0.05.

In the cheese microbial ecology example, there was also significant evidence (adonis test p-value 0.0001) of distance-based dependence among the OTU p-values. Using Hartung's p-value combination method, and controlling the FWER with Hommel's p-value adjustment method, Fig. 3b shows the *SigTree* package results for this cheese microbial ecology example. The branch colors here use the same p-value intervals as in the legend of Fig. 3a, and these colors (and the corresponding branch p-values) were embedded in a resulting Nexus file. In Fig. 3b, this Nexus file from *SigTree* was opened in *FigTree* [4], where rotation, highlighting, and tip label suppression were used to facilitate visualization. The dark red branches correspond to families of OTUs that exhibit an overall significant consensus greater abundance in the presence of Bif-6 than the absence.

While our focus in this presentation is on the general demonstrative nature of the *SigTree* package rather than specific biological conclusions, as one example of biologically relevant outcomes that can be derived from this approach, we comment briefly on the highlighted red branch of Fig. 3b. Many OTUs in this branch represent bacteria that metabolize sulfur in different environments; sulfur metabolism alters cheese flavor beneficially [29]. Even after controlling the family-wise error rate across all branches in the entire phylogenetic tree, there is statistically significant evidence that the presence of Bif-6 results in a consensus increased abundance of this family (or phylogenetic branch) of bacteria. This could in turn induce more sulfur metabolic pathways, and thus provide beneficial flavor changes to the cheese.

## 4 Discussion

*SigTree* provides tools to use results of OTU-level significance tests (with meaningful one-sided p-values) to identify and visualize branches in a phylogenetic tree that are significantly responsive to some treatment intervention or change in environmental conditions. This functionality is not available in any other software package, and *SigTree* does much more than just map data values onto the tree. It provides a convenient interface to a reliable statistical framework allowing meaningful statements regarding significance of the response – not just for the abundance levels of single OTUs, but for entire branches of the phylogenetic tree.

Two methods in the literature that may appear at least superficially similar to *SigTree* are phylofactorization [25] (implemented in R) and Gneiss [26] (implemented in Python). Phylofactorization identifies “sub-groups of taxa [in a given phylogenetic tree] which respond differently to treatment relative to one-another.” [25] This is essentially the same objective as *SigTree*, and the corresponding phylofactorization implementation requires the use of raw data (relative abundance of each tip OTU in each sample) and allows multiple covariates. By comparison, *SigTree* uses tip-level p-values corresponding to the test of a single effect. While this may seem to limit the flexibility of *SigTree*, the opposite is in fact the case – the phylofactorization method is actually designed to test effects only in a multiple regression model, assuming normal data (after an automated isometric log-ratio transform), and not allowing random effects or nesting or repeated measures (which are important characteristics in many study designs, such as the cheese microbial ecology example used here). In contrast, the use of *SigTree* allows (actually requires) users to select an appropriate model for their given study design, including accounting for study-specific data distribution (such as Poisson or negative binomial for count data from a certain high-throughput technology; or choosing a nonparametric test as was done for the mouse gut microbiome study here). While *SigTree* does only look at one effect at a time in a given model, it can look at multiple effects (or contrasts) from a complex model, one at a time. This flexibility in the construction of the *SigTree* approach is intentional, to allow its application in *any* experimental design and with *any* high-throughput technology.

The Gneiss method is designed to “[understand] species distributions across different covariates” [26], and, like the phylofactorization method [25], employs an isometric log-ratio (ILR) transform of the raw OTU relative abundance data. While the Gneiss framework putatively allows for mixed models with multiple covariates, it is left to the user to specify the model (among those available in Python), and Gneiss is constrained by limitations involving the ILR. Specifically, raw data values of zero are problematic for Gneiss's use of the ILR, with the only current solutions being to add a pseudocount or drop certain OTUs. *SigTree* also requires the user to specify the appropriate (and possibly mixed) model for their study design and data, but prior to using the package's functions. This actually allows greater flexibility than Gneiss, such as when it would be meaningful and appropriate to choose a model that explicitly allows (possibly an abundance of) zeros in the raw abundance data, or when a given model is not readily fit using tools in (Gneiss-required) Python. In addition, Gneiss does not include convenient tree-level visualization tools, which are a strength of *SigTree*.

The default multiplicity correction employed by *SigTree* is Hommel [17] (for family-wise error rate control, to allow for stronger conclusion statements). The package also allows control of the false discovery rate using the Benjamini-Yekutieli correction [18]. Both of these corrections were selected for inclusion in *SigTree* because they allow for general positive dependence among tests [18], [27], and it would be reasonable to expect such dependence among the many (and sometimes nested) tests within a tree. However, these corrections do not explicitly account for the dependence possibly induced by the nested structure of the phylogenetic tree. Accounting for dependence among nested or overlapping tests has been previously addressed in the context of gene ontology graphs [28], particularly for false discovery rate control. Adapting such an approach to the phylogenetic tree structure, and addressing family wise error rate control within the nested tree structure, are possibilities for future *SigTree*-related work.

The context of gene ontology graphs actually raises another perspective from which to consider the type of testing done by *SigTree*. The branches (or their corresponding nodes) within a phylogenetic tree are essentially pre-defined groups of OTUs, just as nodes within a gene ontology graph are pre-defined groups of genes. Where the groups of OTUs are based on (estimated) phylogeny, the groups of genes are based on (estimated) common roles in terms of biological processes, molecular functions, or cellular components [31]. The meta-analytic methods employed by *SigTree* in the phylogenetic tree context are similar to those employed by the *mvGST* approach in the gene ontology context [32], [33]. It may be possible to extend or adapt other statistical methods for testing these structured groups (or sets) of genes such as GSEA [34] or ROAST [35], and apply them in the context of a phylogenetic tree. These are also possibilities for future *SigTree-*related work.

Both of the example studies presented here demonstrate a strength of *SigTree* and the statistically powerful meta-analytic methods it employs. After multiple comparison adjustments, it may be that no OTU exhibits an individually significant response to the treatment intervention. For example, all tips are colored grey in Fig. 3, indicating no tip-level significant response to the treatment interventions when the FWER is controlled at 0.05. However, overall tendencies within branches can be detected and legitimately called statistically significant, due to the power of the meta-analytic p-value combination methods employed by *SigTree*. In both of these examples, this statistical power results in the identification (and subsequent visualization in Fig. 3) of branches that do exhibit significant consensus response to the corresponding treatment intervention.

*SigTree*'s reliance on p-values rather than raw data makes this package flexible for any experimental design and high-throughput technology, ensuring its long-term utility to microbial community analysis researchers. *SigTree* is written for the R environment [5], and results can be visualized in R as well as in other programs (such as *FigTree*). *SigTree* is open source, and freely available (with a tutorial vignette demonstrating package code usage) at http://cran.r-project.org/web/packages/SigTree/index.html. The current tutorial vignette is also provided with this manuscript as Supplemental File S1. *SigTree* and its tutorial vignette will be maintained and updated by the corresponding author as future needs evolve.

*SigTree* can help microbial community researchers efficiently make and effectively communicate (in visual form) novel discoveries regarding how the abundance levels of entire families of OTUs (or branches in the phylogenetic tree) are affected by treatment interventions or other environmental changes.

## Appendix A Supplementary Data

Supplemental File S1 *SigTree* tutorial vignette. An overview and demonstration of the main syntax to use the *SigTree* software package, with sample data. Also archived (with possible future updates) at http://cran.r-project.org/web/packages/SigTree/index.html. Supplemental File S1
