# Supplementary material for: SigTree: A Microbial Community Analysis Tool to Identify and Visualize Significantly Responsive Branches in a Phylogenetic Tree
Source: Comput Struct Biotechnol J. 2017 Jul 6;15:372–8. doi: 10.1016/j.csbj.2017.06.002 (PMC5512180; doi:10.1016/j.csbj.2017.06.002)
Supplement: Supplemental File S1 — SigTree tutorial vignette. An overview and demonstration of the main syntax to use the SigTree software package, with sample data. Also archived (with possible future updates) at http://cran.r-project.org/web/packages/SigTree/index.html. [file mmc1.pdf]

# *SigTree* Tutorial Vignette: Identifying and Visualizing Significantly Responsive Branches in a Phylogenetic Tree

John R. Stevens[1], Todd R. Jones[2], and Michael Lefevre[3]

February 7, 2017


1. Associate Professor of Statistics, Dept. of Mathematics and Statistics, Utah State University (`http://www.stat.usu.edu/jrstevens`)

2. Ph.D. Student, Dept. of Economics, Cornell University (`http://www.toddrjones.com`); M.S. Statistics Graduate, Dept. of Mathematics and Statistics, Utah State University

3. USTAR Professor, Dept. of Nutrition, Dietetics, and Food Science; USTAR Applied Nutrition Research Team, Utah State University



**Abstract**

The *SigTree* package provides tools to identify branches in a phylogenetic tree that are significantly responsive to some intervention. Given the phylogenetic tree (as a .tre file) and p-values corresponding to each tip (or OTU, on which the intervention's effect has been tested), these tools obtain p-values for each branch of the tree using a meta-analysis framework. The tree is then visualized with branches and tip labels colored according to significance, with options in place to control the family-wise error rate across the entire tree. These tools also allow additional exploration of the significance results.


**Note:** If you use this package, please cite Stevens *et al.* (2017).

# Contents


**1 Introduction 3**

**2 Getting Started 3**

2.1 Reading in Phylogenetic Tree . . . . . . . . . . . . . . . . . . . . . . . . 4

2.2 Reading in Significance Results . . . . . . . . . . . . . . . . . . . . . . 5

**3 Sample Analysis in SigTree 5**

3.1 Package Features . . . . . . . . . . . . . . . . . . . . . . . . . . . . . 8

3.1.1 Color Palette . . . . . . . . . . . . . . . . . . . . . . . . . . 8

3.1.2 P-value Sidedness . . . . . . . . . . . . . . . . . . . . . . . . 9

3.1.3 Branch Definition and Color . . . . . . . . . . . . . . . . . . . 11

3.1.4 Multiple Testing Adjustment . . . . . . . . . . . . . . . . . . 12

3.2 Exploring Results Further . . . . . . . . . . . . . . . . . . . . . . . . 13

3.2.1 See OTUs In a Given Branch . . . . . . . . . . . . . . . . . . 14

3.2.2 Display Results In FigTree . . . . . . . . . . . . . . . . . . . 15

3.3 Testing on Only a Subset of a Tree . . . . . . . . . . . . . . . . . . . . 16

**4 Misc. Issues 18**

4.1 Visualizing Results of Multiple-Treatment Designs . . . . . . . . . . . . . 18

4.2 Reading in NEXUS Format Tree Files . . . . . . . . . . . . . . . . . . . 18

4.3 Singleton Node Error When Reading in Tree Files . . . . . . . . . . . . . 19

# 1 Introduction

The *SigTree* package provides tools to identify branches in a phylogenetic tree that are significantly responsive to some intervention. It is designed for studies that meet the following criteria:

1. Multiple operational taxonomic units (OTUs) are of interest.
2. A significance test has been performed on each OTU, assessing the degree of its response to some intervention (such as in a treatment vs. control situation).
3. The phylogenetic tree representing the relationships among the OTUs (tips in the tree) is available.
4. The research question is which branches of the phylogenetic tree exhibit an overall significant response to the intervention.

Note that this *SigTree* package does not test the significance of the existence of branches (or how strongly the phylogenetic structure is supported by experimental data or evolutionary theory), but rather tests how each branch responds to some treatment or intervention.

The inputs required for the use of this package are the phylogenetic tree file and a spreadsheet file summarizing the results of the significance test on each OTU. The phylogenetic tree must be provided as a .tre file, in either NEXUS or Newick format. (Basically any tree file exported from FigTree (Rambaut, 2009) can be used.) The spreadsheet file must have one row for each OTU, and two columns – the first recording the name of the OTU, and the second recording the p-value for the OTU's significance test. The OTU names must match in both files.

This tutorial document includes sample R code with a brief discussion of an example.

# 2 Getting Started

First, make sure you are running the most recent version of R. Windows users can use the following link:

`http://cran.r-project.org/bin/windows/base/release.htm`

Next, set the file path to the two input files referred to in Section 1. Let's assume both files are in the same directory. In practice, if the file path to that directory were "C:/folder1/folder2", you'd run this at the command prompt in R:

```
> tre.path <- "C:/folder1/folder2"
```

For this tutorial example, we'll use some sample files that come with the *SigTree* package, so our file path will be different:

```
> tre.path <- system.file("sample", package="SigTree")
```

## 2.1 Reading in Phylogenetic Tree

There is a `sample.tre` file in the *SigTree* package. In this example, there are 100 OTUs, named for convenience t1, ..., t100. This file is in Newick format; files may also be in NEXUS format (see Section 4.2). To read it in and look at this phylogenetic tree, we'll first load the *ape* package (Paradis *et al.*, 2004).

```
> library(ape)
> tree.file <- paste(tre.path,"sample.tre",sep="/")
> tree <- read.tree(tree.file)
> plot(tree,type="fan")
```

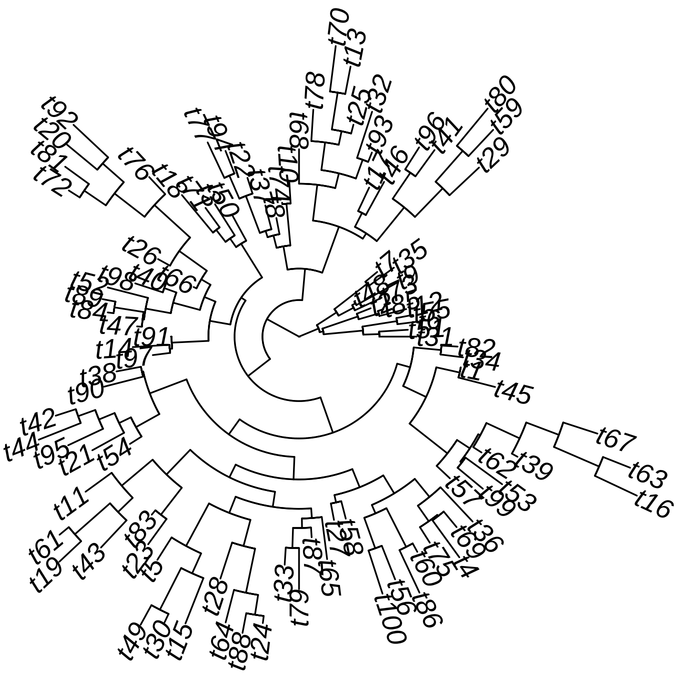

## 2.2 Reading in Significance Results

In the same directory as our `sample.tre` file, there is a `sample.csv` file summarizing the significance test on each of the 100 OTUs. In this sample study, each OTU can be thought of as a strain of bacteria found in the gut of mice. The abundance of each OTU was assessed (using 16s rRNA sequencing) in each of 10 treatment samples (mouse diet T) and 10 control samples (mouse diet C). A Wilcoxon rank-sum test was performed on each OTU individually, corresponding to the hypotheses

$$\begin{aligned} H_0 &: \mu_C = \mu_T \\ &\text{vs.} \\ H_A &: \mu_C < \mu_T, \end{aligned}$$

where $\mu_C$ and $\mu_T$ are the mean abundances of the OTU in the control and treatment populations, respectively.

```
> sig.file <- paste(tre.path,"sample.csv",sep="/")
> frame <- read.csv(sig.file)
> head(frame)
```

```
  OTU      pval
1 t31 0.8862966
2 t51 0.3907253
3  t6 0.1390846
4 t55 0.9905042
5 t12 0.3339162
6 t85 0.3064087
```

Notice that our `sample.csv` file has only two columns – the first for OTU names, and the second for the p-values from the significance test of the OTUs. While this tutorial example focuses on a simple treatment vs. control example, the flexibility of the *SigTree* package allows for any experimental design. The only input required here is this file of p-values for all OTUs of interest. It is the user's responsibility to ensure the appropriateness of statistical methods used the produce these p-values.

# 3 Sample Analysis in SigTree

First, load the *SigTree* package:

```
> library(SigTree)
```

The *SigTree* package has the following four main functions:

- `adonis.tree`
- `plotSigTree`
- `export.inherit`
- `export.figtree`

All four rely on the same main inputs and arguments and are demonstrated in this package tutorial.

First, the `adonis.tree` function can be used to test whether the p-values at the tips are independent or dependent (specifically, whether there are significant differences among p-values, based on the between-tip distances in the phylogenetic tree). This is based on the adonis test (Reiss *et al.* 2010; Anderson, 2001), and returns a p-value for the null "p-values are independent" vs. the alternative "p-value differences are associated with pairwise distances", as follows:

```
> adonis.tree(tree,frame)
```

```
[1] 0.00159984
```

Based on this highly significant result, one could conclude that the p-values for these OTUs are not independent, which dependence can be accounted for in the subsequent meta-analytic steps using Hartung's p-value combination method (see below).

Run the `plotSigTree` function with appropriate arguments:

```
> plotSigTree(tree, frame, test="Hartung")
```

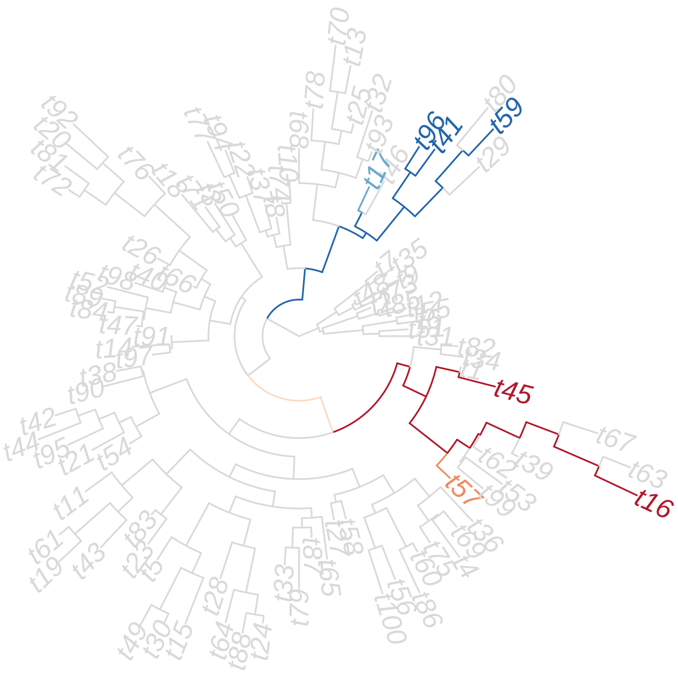


The `plotSigTree` package relies on a meta-analysis framework to systematically combine the p-values for all OTUs within each branch in the phylogenetic tree, resulting in a single p-value for each branch. The resulting plot colors each branch (and tip label) according to its p-value. For much more discussion on this meta-analysis framework and function options, see Jones (2012). Briefly, there are three competing meta-analysis approaches to combine p-values used here:

- Fisher's method (Fisher, 1932). In the context of the current example (where $H_0 : \mu_C = \mu_T$ vs. $H_A : \mu_C < \mu_T$ is tested for each OTU separately in a given branch), this method tests the null "$\mu_C = \mu_T$ for all OTUs in the branch" vs. the alternative "$\mu_C < \mu_T$ for at least one OTU in the branch."

- Stouffer's method (Stouffer *et al.*, 1949; default in *SigTree*). This approach focuses on finding a consensus (Rice, 1990; Whitlock, 2005) among p-values in the branch. It can be considered to test the null "$\mu_C = \mu_T$ for all OTUs in the branch" vs. the alternative "there is a consensus among OTUs in the branch that $\mu_C < \mu_T$." Stouffer's method implicitly assumes independent p-values.

- Hartung's method (Hartung, 1999). This is a generalized version of Stouffer's method, and allows dependence among p-values. Hartung assumes a constant (and nonnegative) correlation among all pairs of p-values, and shows that results are stable even when the correlation is non-constant.

Because the intent of the *SigTree* package is to aid in identifying "which branches of the phylogenetic tree exhibit an overall significant response to the intervention" (Section 1), Stouffer's method is the recommended (and default) p-value combination method. In the event that `adonis.tree` shows significant evidence of dependence among p-values, Hartung's method is recommended (as a generalized version of Stouffer's method). Fisher's method can be used instead using the `test` argument in the `plotSigTree` function.

## 3.1 Package Features

### 3.1.1 Color Palette

The default color palette (for one-sided p-values as in the current example) is from dark red for low p-values to dark blue for high p-values. The following example shows how to change this palette and also make the tree more clear. A legend can also be added to highlight conclusions – here, that OTUs in a particular branch in the tree (dark red, with very low p-values) tend to be overall less abundant in diet C than in diet T, while those in another branch (dark blue, with very high p-values) tend to be overall more abundant in diet C than in diet T.

```
> library(RColorBrewer)
> RdBu <- brewer.pal(7, "RdBu")
> RdBu[4] <- brewer.pal(7, "Greys")[3]
> plotSigTree(tree, frame, test="Hartung",pal=RdBu, tip.label.size=.8,
+             edge.width=2)
> legend("topright",c("C > T","C < T"),cex=1.5,text.col=RdBu[c(7,1)],bty="n")
```

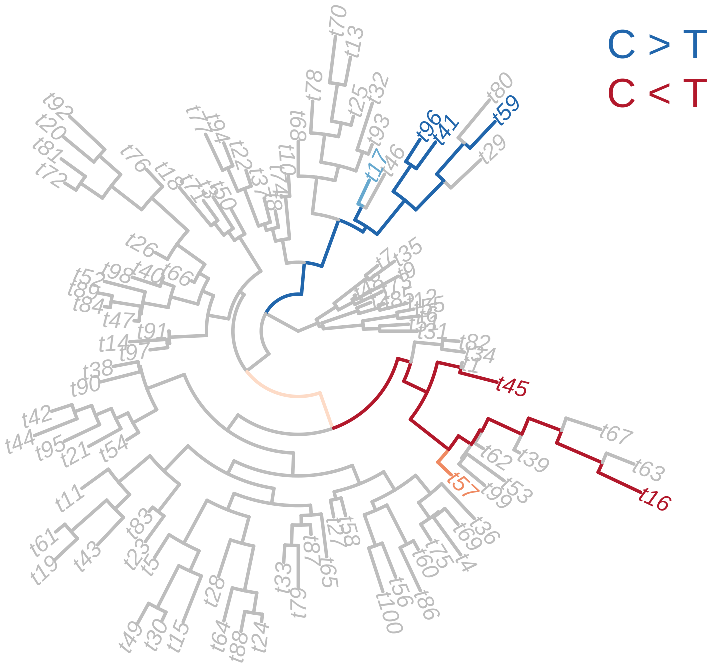


For one-sided tests, the default p-value cutoffs for the various colors are 0.01, 0.05, 0.10, 0.90, 0.95, and 0.99. These can be changed using the `p.cutoffs` argument of the `plotSigTree` function.

#### 3.1.2 P-value Sidedness

Because of the one-sided alternative in the current example ($H_A$: $\mu_C < \mu_T$), it is worth mentioning here that the resulting one-sided p-values can be compared to two different significance thresholds – one near zero and another near one. P-values below the smaller threshold (say $\alpha_0 = 0.05$) provide evidence to support $\mu_C < \mu_T$. On the other hand, p-values above the upper threshold (say $1 - \alpha_1 = 0.95$) provide evidence to support the other direction, $\mu_C > \mu_T$. Whatever thresholds $\alpha_0$ and $\alpha_1$ are used, the actual Type I error rate is $\alpha = \alpha_0 + \alpha_1$. One of the reasons that Stouffer's method is preferred to Fisher's in *SigTree* is that Stouffer's approach symmetrically preserves this sidedness interpretation while Fisher's does not.

In cases where a two-sided test was made on each OTU, then the `side` argument of

`plotSigTree` can be used to specify the situation. (The following code is not run here.)

```
> plotSigTree(tree, frame, test="Hartung", side=2)
```

In such a case, there is only one significance threshold $\alpha$, and p-values below that threshold provide evidence to support the (two-sided) alternative.

Returning to the current example (with its one-sided alternative), a legend can be added to the plot to communicate the translation of color to p-value range:

```
> plotSigTree(tree, frame, test="Hartung", pal=RdBu, tip.label.size=.8,
+             edge.width=2)
> p.cut.leg <- c("0.00 - 0.01","0.01 - 0.05","0.05 - 0.10",
+                "0.10 - 0.90","0.90 - 0.95","0.95 - 0.99","0.99 - 1.00")
> legend("topright",rev(p.cut.leg),text.col=rev(RdBu), bty="n", cex=1)
```

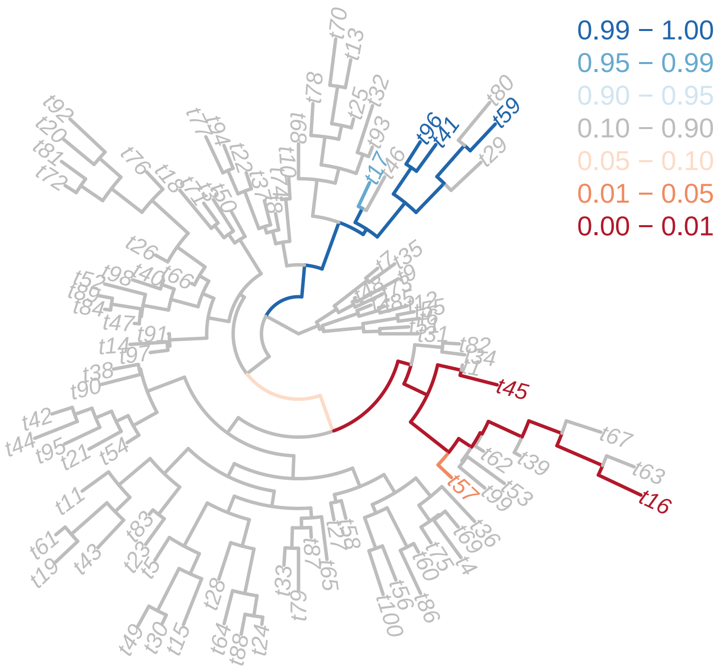

If package users have more traditional two-sided p-values, and also have (for each OTU) a mean difference (or other value indicating direction of difference as positive or negative), the function `p2.p1` can be used to convert to one-sided p-values.

### 3.1.3 Branch Definition and Color

In the *SigTree* package, branches within the phylogenetic tree are colored based on their p-value. A "branch" can be defined in one of two ways, using the `branch` argument of the three main *SigTree* functions. While this argument will not affect the meta-analysis p-values obtained for groups of OTUs, it will affect which part of the phylogenetic tree is colored based on these p-values.

- When `branch="edge"` (the default in package version 1.2 and later), each tip label and the single edge leading to it (away from the root) will be colored based on the tip OTU's p-value. Neighboring tips' edges are not necessarily the same color because the tip OTUs could have different p-values. Neighboring tips' edges join at an interior node, and the single edge leading to that node (from the root direction) is colored based on the combined p-values of the OTUs below that edge and interior node (away from the root). The two edges from any given interior node (away from the root) would not necessarily be the same color, because they include different sets of tips. Thus the default definition of "branch" is the collection of all tips below an edge (away from the root) in the phylogenetic tree.
- When `branch="node"`, a "branch" is instead defined as the collection of all tips below the interior node in each of the two edges extending from the interior node (away from the root). For example, in the following plot, the highest-level red interior node has two edges – one (on the left) leading to a large collection of grey sub-branches, and the other (on the right) leading to a collection of largely red sub-branches. Both of these edges from that interior node are colored according to the p-value from Stouffer's combination of the p-values of all tips (OTUs) below that interior node (where the two edges meet).

Compare the following plot (where `branch="node"`) with the final plot of Section 3.1.2 (where by default `branch="edge"`). See Section 3.2.1 for a discussion of how to explore OTUs within a given branch.

```
> plotSigTree(tree, frame, test="Hartung", pal=RdBu, tip.label.size=.8,
+              edge.width=2, branch="node")
> p.cut.leg <- c("0.00 - 0.01","0.01 - 0.05","0.05 - 0.10",
+                 "0.10 - 0.90","0.90 - 0.95","0.95 - 0.99","0.99 - 1.00")
> legend("topright",rev(p.cut.leg),text.col=rev(RdBu), bty="n", cex=1)
```

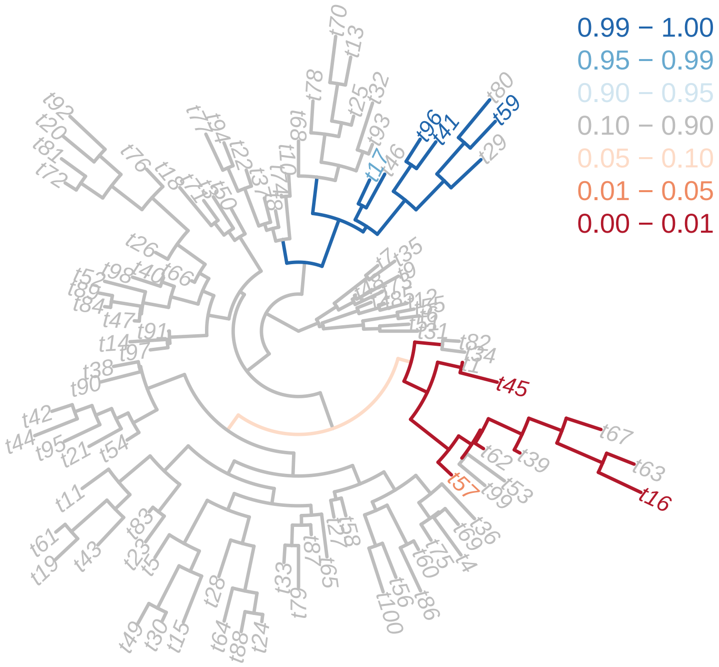


### 3.1.4 Multiple Testing Adjustment

In a phylogenetic tree with $n$ OTUs (tips), there will be $n-1$ interior branches at which p-value combination will be done. This will result in a total of $2n-1$ p-values (one for each branch). In order for a tree-wide significance threshold $\alpha$ (see discussion in Section 3.1.2) to be meaningful, some kind of adjustment must be made to account for this multiple testing problem, while allowing for dependency among p-values (as the interior branch p-values are not independent of the tip p-values). Any of the adjustments implemented in the `p.adjust` function can be called by the `method` argument in the functions of the *SigTree* package:

```
> p.adjust.methods

[1] "holm"       "hochberg"   "hommel"     "bonferroni" "BH"
[6] "BY"         "fdr"        "none"
```

The default in the *SigTree* package is to make Hommel's correction (Hommel, 1988) because, among these options in `p.adjust`, it provides the best strong control of the family-wise error rate under dependency (Blakesley *et al.*, 2009). If instead the user wanted to control the false discovery rate while allowing dependency among p-values, the adjustment of Benjamini and Yekutieli (2001) could be used:

```
> plotSigTree(tree, frame, test="Hartung", method="BY")
```

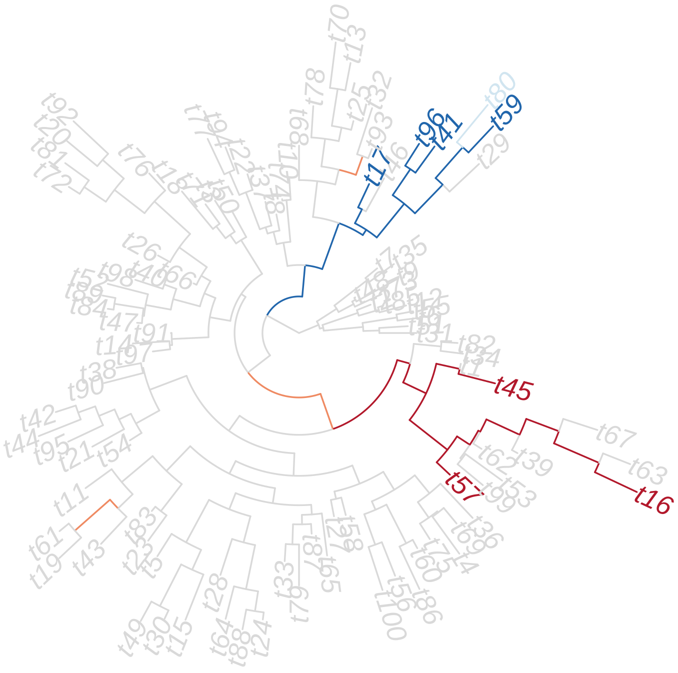


## 3.2 Exploring Results Further

Once the phylogenetic tree has been visualized with branches and tip labels colored to represent significance, the results can be explored in greater detail, both in R as well as in FigTree.

### 3.2.1 See OTUs In a Given Branch

In the figure at the end of Section 3.1.2, we can focus on the red branch at the right side. Each interior branch of the tree has a number, and these numbers can be seen by using the `branch.label=TRUE` option (with label sizes adjustable):

```
> plotSigTree(tree, frame, test="Hartung", pal=RdBu, tip.label.size=.8,
+             edge.width=2, branch.label=TRUE, branch.label.size=.75)
> edgelabels(edge=176,frame="circ",bg="yellow",cex=.8)
```

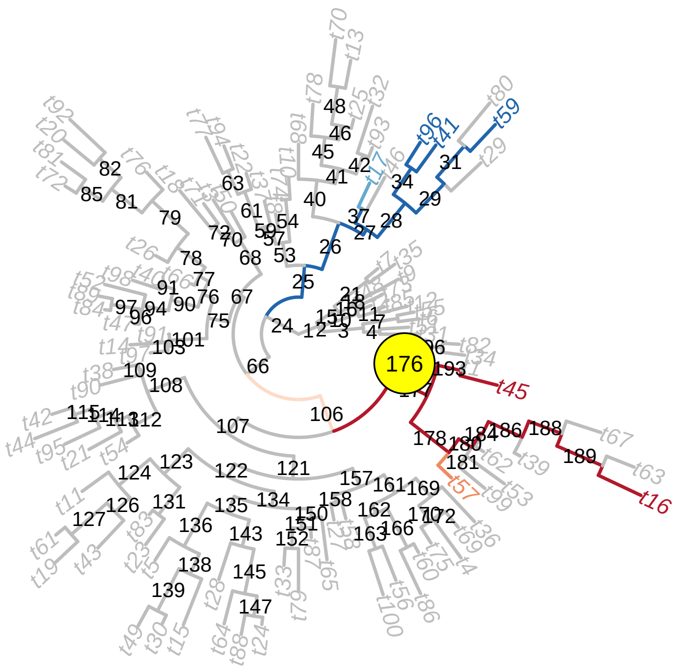

Based on the branch numbering, suppose we wanted to explore branch 176 further. This can be done in two ways, both using the `export.inherit` function of *SigTree*. First, we could create a .csv file summarizing the p-value and member OTUs of each branch in the tree (this code is not run here):

```
> export.inherit(tree, frame, test="Hartung", file="sampleInherit.csv")
```

Alternatively, we could look at the contents of this file in R by using the `frame=TRUE` option:

```
> temp <- export.inherit(tree, frame, test="Hartung", frame=TRUE)
```

This `temp` object is a data frame, with a row for each branch (or tip) in the tree, and columns for the branch number (or tip name), p-value, and members. Branch 176 can be explored:

```
> br176 <- temp[temp$Branch=="176",]
> t <- !is.na(br176[1,])
> br176[1,t]

    Branch Hartung's p-value  X1  X2  X3  X4  X5  X6  X7  X8  X9 X10 X11 X12
189    176      1.685006e-11 t57 t99 t53 t62 t39 t16 t63 t67 t45  t1 t34 t82
```

The OTUs in branch 176 are t57, t99, t53, t62, t39, t16, t63, t67, t45, t1, t34, and t82. Overall, this family of OTUs has significant consensus (Hommel-adjusted p-value ≈ 1.7e-11) that $\mu_C < \mu_T$.

This result can be compared with the larger (and less significant) family of OTUs corresponding to branch 106:

```
> br106 <- temp[temp$Branch=="106",]
> t <- !is.na(br106[1,])
> br106[1,t]

    Branch Hartung's p-value  X1  X2  X3  X4  X5  X6  X7  X8  X9 X10 X11 X12
154    106        0.09452722 t38 t90 t42 t44 t95 t21 t54 t11 t61 t19 t43 t83
    X13 X14 X15 X16 X17 X18 X19 X20 X21 X22 X23 X24 X25 X26 X27  X28 X29 X30
154 t23  t5 t49 t30 t15 t28 t64 t88 t24 t33 t79 t87 t65 t27 t58 t100 t56 t86
    X31 X32 X33 X34 X35 X36 X37 X38 X39 X40 X41 X42 X43 X44 X45 X46 X47
154 t60 t75  t4 t69 t36 t57 t99 t53 t62 t39 t16 t63 t67 t45  t1 t34 t82
```

### 3.2.2 Display Results In FigTree

The p-values and corresponding branch colors generated by the statistical methods of *SigTree* can be exported in a format that can be viewed in FigTree (Rambaut, 2009) by using the `export.figtree` function (with default `branch="edge"`):

```
> library(phyext2)
> export.figtree(tree, frame, test="Hartung", pal=RdBu, file="sigsample.tre")
```

The resulting `sigsample.tre` file will be in NEXUS format. It can be opened and explored in FigTree. For example, the p-values of a selected branch can be viewed in FigTree by selecting "Annotate" and then "Annotation = P-value". The following screenshot shows a sample result for branch 106 (showing the same combined p-value as at the end of Section 3.2.1):

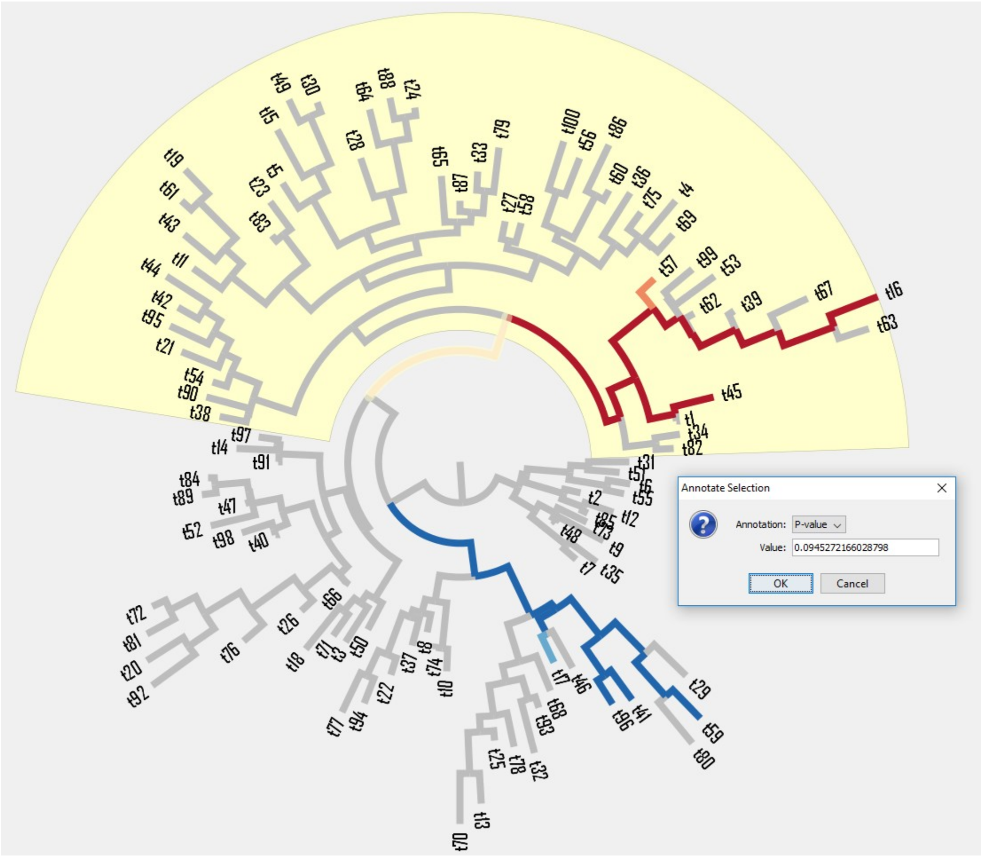


Note that the root edge visible in the screenshot above can also be plotted in R using the `root.edge=TRUE` option in the `plotSigTree` function.

## 3.3 Testing on Only a Subset of a Tree

If p-values are available for only a subset of the tips in a given phylogenetic tree, or if there is some filter that is applied to restrict attention to such a subset, the tree can be "pruned" to include only those tips. This first uses the `prune_taxa` function of the *phyloseq* package to obtain the new tree:

```
> keep.taxa <- c("t57","t99","t53","t62","t39","t16",
+                 "t63","t67","t45","t1","t34","t82")
```

```
> library(phyloseq)
> new_tree <- prune_taxa(keep.taxa, tree)
```

In the functions of the *SigTree* package, the data frame containing the p-values for each OTU must have exactly the same OTU names as the tips in the tree:

```
> t <- is.element(frame$OTU, keep.taxa)
> new_frame <- frame[t,]
> plotSigTree(new_tree, new_frame, test="Hartung", pal=RdBu, tip.label.size=1.5,
+              edge.width=4)
```

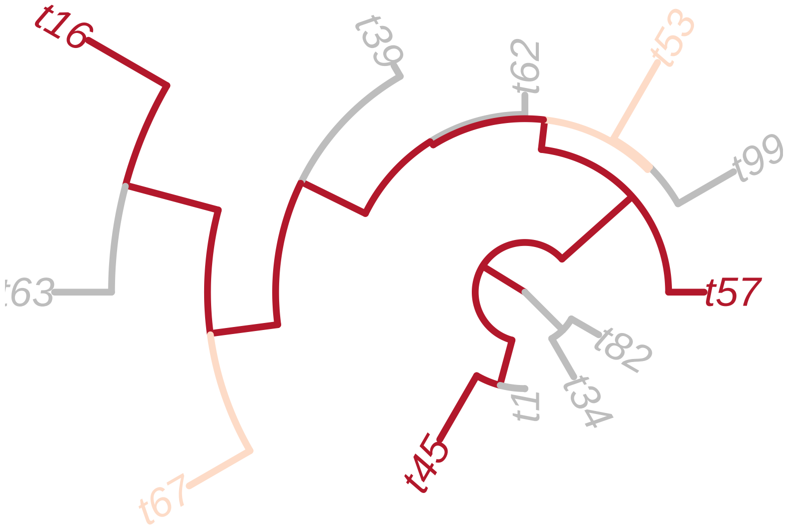

# 4 Misc. Issues

## 4.1 Visualizing Results of Multiple-Treatment Designs

Some users of *SigTree* have inquired regarding multiple-treatment designs, rather than two-treatment designs. For example, consider a design with three treatment groups (T1, T2, T3), and suppose the researcher is interested in visualizing branches in the phylogenetic tree that respond differently to the various treatments.

For each OTU, a single p-value for the global null ($H_0$: $\mu_{T1} = \mu_{T2} = \mu_{T3}$) can be obtained, and *SigTree* tools can help visualize which branches of the phylogenetic tree exhibit *any* differences among treatments. However, to visualize the specifics of those differences, all pairwise comparisons among treatments must be considered separately. This is because a branch could be significantly different between T1 and T2, but also significantly different between T2 and T3, and also between T1 and T3. In such a case, three colors (one for each pairwise comparison) could be overlayed on the tree, but that would quickly cause confusion, especially for larger branches, considering how SigTree combines p-values (for a single comparison) within branches to get a branch-level p-value (for that same single comparison). Instead, a separate colored tree is needed for each pairwise comparison (T1 vs. T2, T2 vs. T3, and T1 vs. T3).

While it might seem awkward to have multiple displayed trees, this approach does give the most direct (and honest) visualization of the specific differences with multiple treatment groups.

## 4.2 Reading in NEXUS Format Tree Files

The .tre file created by the `export.figtree` function in Section 3.2.2 can be read back in to R, but using the `read.tree` function will result in an error:

```
> sig_tree <- read.tree("sigsample.tre")

Error in if (tp[3] != "") obj$node.label <- tp[3] :
  missing value where TRUE/FALSE needed
```

This error is due to the NEXUS format of the .tre file. Use `read.nexus` to read in this format of .tre file:

```
> sig_tree <- read.nexus("sigsample.tre")
```

## 4.3 Singleton Node Error When Reading in Tree Files

If a tree has a singleton node, it cannot be read in to R in the usual way. There is such a `singletonsample.tre` file with the *SigTree* package.

```
> singletontree.file <- paste(tre.path,"singletonsample.tre",sep="/")
> tree <- read.tree(singletontree.file)
Error in read.tree(singletontree.file) :
  The tree has apparently singleton node(s): cannot read tree file.
  Reading Newick file aborted at tree no. 1
```

This problem is not exactly a *SigTree* issue, but since we have experienced it with sample analyses, we wanted to post its solution in case package users experience it. Briefly, the problem is an unexpected "(" at the beginning and "num:num)" at the end of the .tre file. A regular expressions solution for this problem is presented here:

```
> t1 <- read.table(singletontree.file)
> # remove initial (
> t1.split <- strsplit(as.character(t1$V1),"")[[1]]
> t2 <- paste(t1.split[-1],collapse="")
> # remove last ) and the num:num preceding it
> t2.split <- strsplit(t2,")")[[1]]
> t2.len <- length(t2.split)
> t3.split <- t2.split[c(1:(t2.len-2),t2.len)]
> t3 <- paste(t3.split,collapse=")")
> # write to file (fixed now) and read in again
> newtree.file <- paste(tre.path,"fixedsinglesample.tre",sep="/")
> write.table(t3,file=newtree.file, quote=F, col.names=F, row.names=F)
> t4 <- read.tree(newtree.file)
```

This `fixedsinglesample.tre` file will contain the exact same structure as in the original `singletonsample.tre` file without the problematic singleton node.

# References


[1] Anderson, M.J. (2001) "A new method for non-parametric multivariate analysis of variance," *Austral Ecology* 26:32-46.

[2] Blakesley R.E., Mazumdar S., Dew M.A., Houck P.R., Tang G., Reynolds C.F., and Butters M.A. (2009), "Comparisons of Methods for Multiple Hypothesis Testing in Neurophsychological Research," *Neuropsychology* 23(2):255-264.

[3] Fisher R.A. (1932), *Statistical Methods for Research Workers*, 4th ed. Oliver and Boyd, Edinburgh.

[4] Hartung J. (1999) "A Note on Combining Dependent Tests of Significance," *Biometrical Journal* 41(7):849-855.

[5] Hommel G. (1988), "A Stagewise Rejective Multiple Test Procedure Based on a Modified Bonferroni Test," *Biometrika* 75:383-386.

[6] Jones T.R. (2012), "SigTree: An Automated Meta-Analytic Approach to Find Significant Branches in a Phylogenetic Tree," M.S. Thesis, Utah State University, Department of Mathematics and Statistics. http://digitalcommons.usu.edu/etd/1314

[7] Paradis E., Claude J., and Strimmer K. (2004), "APE: Analyses of Phylogenetics and Evolution in R Language," *Bioinformatics* 20:289-290.

[8] Rambaut A. (2009), "FigTree," `http://tree.bio.ed.ac.uk/software/figtree`

[9] Reiss P.T., Stevens M.H.H., Shehzad Z., Petkova E., and Milham M.P. (2010) "On Distance-Based Permutation Tests for Between-Group Comparisons," *Biometrics* 66:636-643.

[10] Rice W.R. (1990), "A Consensus Combined P-Value Test and the Family-Wide Significance of Component Tests," *Biometrics* 46(2):303-308.

[11] Stevens J.R., Jones T.R., Lefevre M.L., Ganesan B., and Weimer B.C. (2017), "SigTree: a microbial community analysis tool to identify and visualize significantly responsive branches in a phylogenetic tree," manuscript in preparation.

[12] Stouffer S.A., Suchman E.A., DeVinney L.C., Star S.A., and Williams Jr. R.M. (1949), *The American Soldier. Vol. 1, Adjustment During Army Life.* Princeton University Press, Princeton.

[13] Whitlock C.M. (2005), "Combining Probability from Independent Tests: The Weighted Z-Method is Superior to Fisher's Approach," *Journal of Evolutionary Biology* 18(5):1368-1373.
